# Prognostic Impact of Induced Natriuresis in Acute Decompensated Heart Failure and Its Association with Intraabdominal Pressure and Other Congestion Markers: A Multimodal Approach to Congestion Assessment

**DOI:** 10.3390/jcm13041053

**Published:** 2024-02-12

**Authors:** Silvia Crespo-Aznarez, Amelia Campos-Saenz de Santamaría, Marta Sánchez-Marteles, Claudia Josa-Laorden, Fernando Ruiz-Laiglesia, Beatriz Amores-Arriaga, Vanesa Garcés-Horna, Ruben Tejel-Puisac, María Angel Julián-Ansón, Ignacio Giménez-López, Juan Ignacio Pérez-Calvo, Jorge Rubio-Gracia

**Affiliations:** 1 Internal Medicine Department, Hospital Clínico Lozano Blesa, 50009 Zaragoza, Spain; silviacrespoaz@gmail.com (S.C.-A.); ameliacampos97@gmail.com (A.C.-S.d.S.); marta.sanchez15@yahoo.es (M.S.-M.); fruizl@unizar.es (F.R.-L.); vanesa_garces@hotmail.com (V.G.-H.); 2 Aragon Health Research Institute (IIS Aragon), 50009 Zaragoza, Spain; claudiajosa@gmail.com (C.J.-L.); amoresarriaga@yahoo.es (B.A.-A.); igimenez@unizar.es (I.G.-L.); jiperez@unizar.es (J.I.P.-C.); 3 Internal Medicine Department, Hospital Royo Villanova, 50015 Zaragoza, Spain; 4 Emergency Department, Hospital Clínico Lozano Blesa, 50009 Zaragoza, Spain; 5 Clinical Biochemistry Deparment, Hospital Clínico Lozano Blesa, 50009 Zaragoza, Spain; rtejel@salud.aragon.es (R.T.-P.); majuliana@salud.aragon.es (M.A.J.-A.); 6 School of Medicine, University of Zaragoza, 50009 Zaragoza, Spain; 7 Aragon’s Institute of Health Sciences (IACS), 50009 Zaragoza, Spain

**Keywords:** heart failure, congestion, natriuresis, intraabdominal pressure, point-of-care ultrasound, diuretic response

## Abstract

Background: Congestion is an essential issue in patients with heart failure (HF). Standard treatments do not usually achieve decongestion, and various strategies have been proposed to guide treatment, such as determination of natriuresis. After starting treatment with loop diuretics, we postulate that initial natriuresis might help treatment titration, decongestion, and improve prognosis. Methods: It was a prospective and observational study. Patients admitted with the diagnosis of HF decompensation were eligible. An assessment of congestion was performed during the first 48 h. Results: A total of 113 patients were included. A poor diuretic response was observed in 39.8%. After the first 48 h, patients with a greater diuretic response on admission (NaU > 80 mmol/L) showed fewer pulmonary b lines (12 vs. 15; *p* = 0.084), a lower IVC diameter (18 mm vs. 22 mm; *p* = 0.009), and lower IAP figures (11 mmHg vs. 13 mmHg; *p* = 0.041). Survival analysis tests demonstrated significant differences showing a higher proportion of all-cause mortality (ACM) and HF rehospitalization in the poor-diuretic-response group (log-rank test = 0.020). Conclusions: Up to 40% of the patients presented a poorer diuretic response at baseline, translating into worse outcomes. Patients with an optimal diuretic response showed significantly higher abdominal decongestion at 48 h and a better prognosis regarding ACM and/or HF rehospitalizations.

## 1. Introduction

Congestion is the main therapeutic target in patients with HF who are admitted with acute symptoms [1]. Endovenous (e.v.) loop diuretics are the gold-standard treatment to improve clinical congestion [2,3]. However, this strategy is inefficient as several studies have shown that around 30–40% of acute HF patients have signs of persistent congestion at discharge, leading to worse outcomes [4,5,6]. Hence, alternative therapeutical strategies have been proposed to improve congestion removal, achieve an efficient diuretic response, and improve outcomes. In this sense, serum biomarkers such as the amino-terminal fragment of pro-brain natriuretic peptide (NT-proBNP) [7], Carbohydrate antigen 125 (CA125) [8], or point-of-care ultrasound (POC) [9] have been proposed as additional tools, besides clinical signs of congestion, to guide e.v. diuretics during acute decompensated heart failure (ADHF), and the results have been promising [8,10].

The analysis of initial natriuresis after the first doses of e.v. furosemide has been demonstrated to have prognostic implications in ADHF [11,12]. Furthermore, some studies suggest that it could help clinicians adjust decongestive treatment early after admission for ADHF. Testani et al. [13,14] demonstrated that urinary sodium concentrations in a random sample after the two hours of e.v. loop diuretic predicts total diuresis [13] during the following six hours, as well as total natriuresis, diuresis, and prognosis [14,15]. Even more, induced natriuresis during the initial 24 h indicates a diuretic response and prognosis that is better than diuresis alone [16]. Natriuresis is becoming central for assessing diuretic response in patients with ADHF, and, along with the glomerular filtration rate (GFR), it allows for stratification of vital prognosis better than these two parameters on their own [17]. However, natriuresis-guided therapy has not yet demonstrated improvement regarding all-cause mortality or first-heart-failure rehospitalizations [18,19]. Therefore, a multimodal approach to evaluating these patients, including other parameters, might improve the assessment and management of decompensation episodes.

We hypothesized that initial natriuresis after starting e.v. furosemide has prognostic implications in ADHF patients due to its link to tissular and intravascular decongestion. The main objectives of this study are (1) to analyze the prognostic impact of baseline natriuresis. (2) To analyze the association of natriuresis with markers of congestion. (3) To analyze the association between natriuresis and abdominal congestion through intraabdominal pressure (IAP).

## 2. Materials and Methods

Study population: Observational and retrospective analyses were carried out at the Internal Medicine Ward of a tertiary hospital, between 2016 and 2023. Inclusion criteria were (1) Age > 18 age years old. (2) NT-proBNP > 1000 pg/mL. (3) Symptons (dyspnea in NYHA functional class II, III, IV) and/or signs of congestion (edema, ascites, jugular engorgement, lung crackles, or pulmonary congestion signs on chest X-ray) due to HF. (4) Informed consent signed. Exclusion criteria were (1) intensive-care previous admission. (2) Impaired cognitive or functional status. (3) End-stage kidney disease (CrCl < 10 mL/min, dialysis and/or renal transplant) [20]. (4) Advanced Chronic obstructive pulmonary disease (COPD) is defined as FEV1 < 30%. Medical data, including previous antecedents, physical examination, and vital signs, were recorded during the first 48 h of admission.

Multimodal assessment of congestion: Tissular lung congestion was detected through the presence of b-lines [21]. We used a portable ultrasound system (Lumify, Philliphs©) and a sectorial probe for explorations. A 12-zone protocol has been previously validated to evaluate patients with acute respiratory failure performing lung ultrasound [22]. Nevertheless, previous studies have shown that an 8-zone protocol is as accurate as the former option [23]. In our examination, 8 thoracic quadrants ((4 right zones and 4 left zones) were explored by trained staff. If 3 or more B-lines were identified in each field, it was considered a positive result. Total b-lines detected were registered at baseline and after 48 h of admission. Intravascular congestion was quantified by analyzing inferior vena cava (IVC) morphology. The portable ultrasound device Lumify (Phillips©) was again used to measure IVC diameter at baseline and after 48 h of e.v. diuretic treatment. The subcostal view allows the estimation of the IVC diameter, and it should be evaluated proximal to the entrance of hepatic veins into the IVC. The collapsibility of IVC was also estimated with a cut-off of < 50% as pathological [24]. The deadline to perform these explorations (lung and IVC ultrasound) was six hours after the first morning e.v. diuretic dose.

Intraabdominal pressure measurement: Intra-abdominal pressure was measured using the vesical catheterization technique using pre-specified equipment designed for this purpose (Unometer Abdo-pressure©). Urine catheters are commonly used during episodes of AHF and are considered a low-risk procedure. A bladder catheter has to be placed in every case; those patients who had not had a medical indication for the utilization of a catheter before their study inclusion would be offered to use one. This technique consists of placing a small volume of saline solution (25 mL) through a closed system with a water column that registers IAP in real time. This technique has been previously validated in HF patients [25].

Laboratory analysis: A complete blood test analysis was performed on the first morning after admission. The estimated glomerular filtration rate (eGFR) was calculated with the Chronic Kidney Disease Epidemiology Collaboration Creatinine formula (CKD-EPI-creatinine). NT-proBNP and CA125 concentrations were determined with specific laboratory kits (Roche Elecsys^®^ NT-proBNP assay; Roche Elecsys^®^CA 125 assay). The urinary natriuresis was determined from urinary spot samples collected between two and three hours after the administration of the first bolus of e.v. morning furosemide. The bladder was emptied before the application of furosemide.

Statistical analysis: Continuous variables were expressed as the mean (±standard deviation) or median (Interquartile range) as appropriate. Qualitative variables were expressed as a percentage. Baseline patient characteristics were stratified based on a cut-off point selected from previous studies (NaU ≤ 80 mmol/L vs. NaU > 80 mmol/L) [26] and compared using the Student’s t-test or Mann–Whitney U test for continuous variables and chi-square test for categorical variables. The primary endpoint of the study was the composite of all-cause mortality (ACM) and/or HF readmissions at 90 days. As secondary objectives, ACM at 90 days, HF rehospitalizations after 90 days, and mean length of stay were analyzed separately. Kaplan–Meier survival curves were compared using the log-rank test. The Cox regression model was used to identify potential predictors of the primary endpoint (ACM and/or HF rehospitalizations at 90 days). First, the candidate variables were chosen using a univariate analysis, selecting as possible independent predictors those variables with a *p*-value < 0.100. The multivariate analysis was carried out in steps, conditionally, and backwards. Continuous variables were transformed with fractional polynomials if needed. The confidence intervals were 95%, establishing statistical significance when *p* < 0.005. All analyses were carried out using SPSS (Statistical Package for the Social Sciences; version 24) and JAMOVI. The study was carried out in compliance with the recommendations contained in the international declaration of Helsinki. The study was approved by the Aragon HealthResearch Ethics Committee (9 September 2015; Ref. C.P.-C.I. PI15/0227).

This manuscript has been elaborated considering the STROBE checklist guidelines [27].

## 3. Results

**Figure 1:**
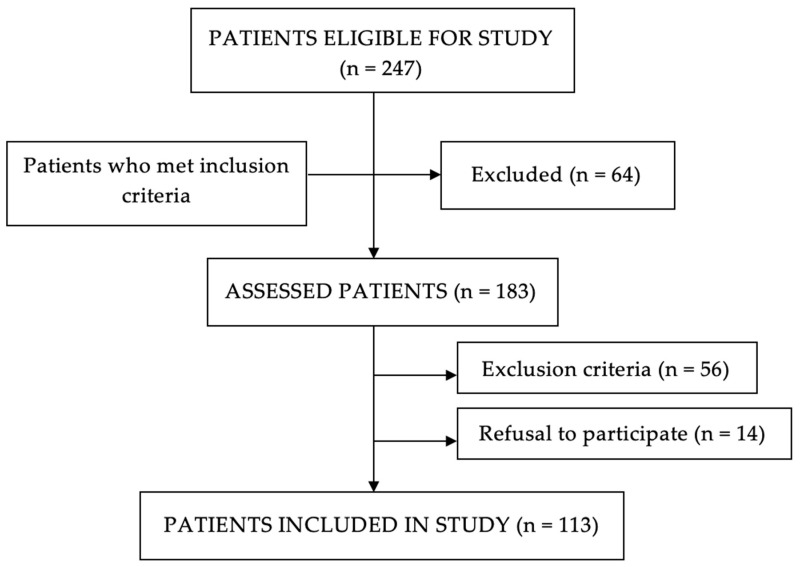
Flow chart of patient selection.

Inclusion and exclusion criteria were applied after selecting eligible patients for the study. Patients who met those criteria were included in the study. A total of 113 patients were recruited (Figure 1).

### 3.1. Baseline Characteristics

The mean age was 81.7 ± 8.3 years; 54% were women, and 61.1% of the sample had HF with preserved ejection fraction (HFpEF). The most frequent comorbidities were arterial hypertension (81.4%), atrial fibrillation (66.4%), dyslipidemia (54.9%), and diabetes mellitus (36.3%). Impaired renal function (eGFR < 60 mL/min/1.73 m^2^) was present in 64% of patients, and of these, 49% presented with an eGFR between 59 and 30 mL/min. The percentage of use of angiotensin-converting enzyme inhibitors (ACEI), angiotensin receptor antagonists, or neprilysin inhibitors (ACEI/ARB/ARNI) was approximately 60%; this percentage was similar for β-blockers use. On the other hand, 21.2% were being treated with mineralocorticoid receptor blockers (MRB), and approximately 10% had previously received treatment with sodium-glucose cotransporter type 2 inhibitors (SGLT2i). (Table 1).

Baseline characteristics according to initial spontaneous urine sodium concentrations (NaU) after the first e.v. dose of furosemide are shown in Table 1. A poor diuretic response (NaU ≤ 80 mmol/L) was observed in 39.8% of patients. These patients had higher NT-proBNP concentrations on admission (6227 pg/mL vs. 4113 pg/mL; *p* = 0.056), lower natremia (138 mmol/L vs. 141 mmol/L; *p* < 0.001), and lower chloremia (97 mmol/L vs. 100 mmol/L; *p* = 0.002). (Table 1).

### 3.2. Multimodal Assessment of Congestion and Intraabdominal Pressure

Intraabdominal pressure was registered in 57 patients. Baseline multimodal assessment of congestion and IAP did not differ between both groups at admission. However, after the first 48 h of admission, patients with a greater diuretic response on admission (NaU > 80 mmol/L) showed a trend of having fewer pulmonary b-lines (12 vs. 15; *p* = 0.084), a lower IVC diameter (18 mm vs. 22 mm; *p* = 0.009), and lower IAP values (11 mmHg vs. 13 mmHg; *p* = 0.041). In addition, urinary sodium concentrations in patients with the greatest diuretic response continued to be significantly higher after the first 48 h (84 mmol/L vs. 75 mmol/L; *p* = 0.042). (Table 2).

### 3.3. Outcomes

**Figure 2:**
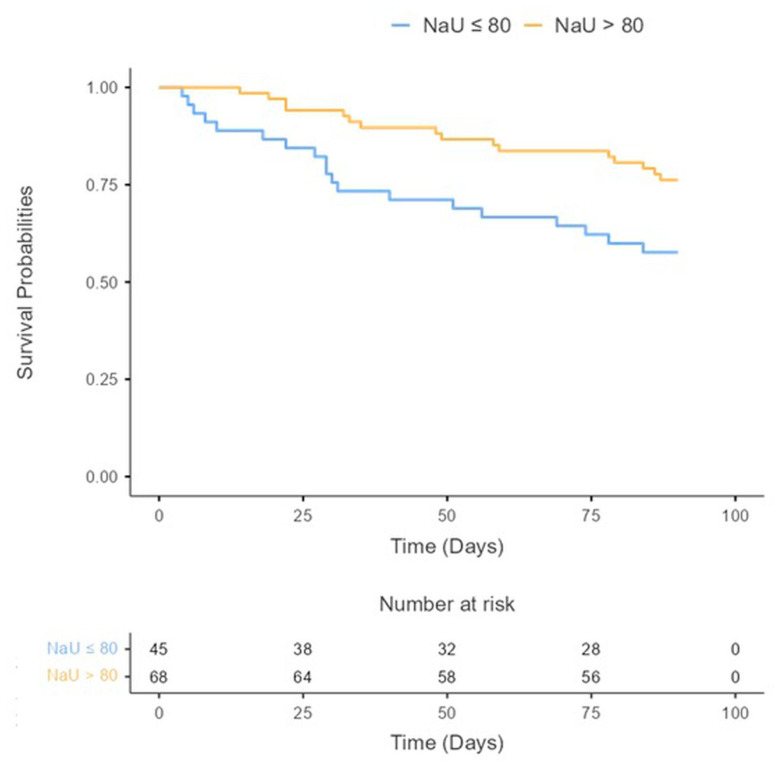
All-cause mortality and/or HF rehospitalization during 90 days after discharge according to baseline urinary sodium concentrations.

During the follow-up period (90 days), a total of 19 patients (16.8%) died, 22 patients (18.6%) were readmitted for HF, and a total of 35 (31%) achieved the primary endpoint (ACM and/or HF rehospitalization at 90 days). Kaplan–Meier curves and a log-rank test showed significant differences between groups. Patients with a poor diuretic response (NaU ≤ 80 mmol/L) experienced a higher proportion of events (Log-rank test = 0.020) (Figure 2).

**Figure 3:**
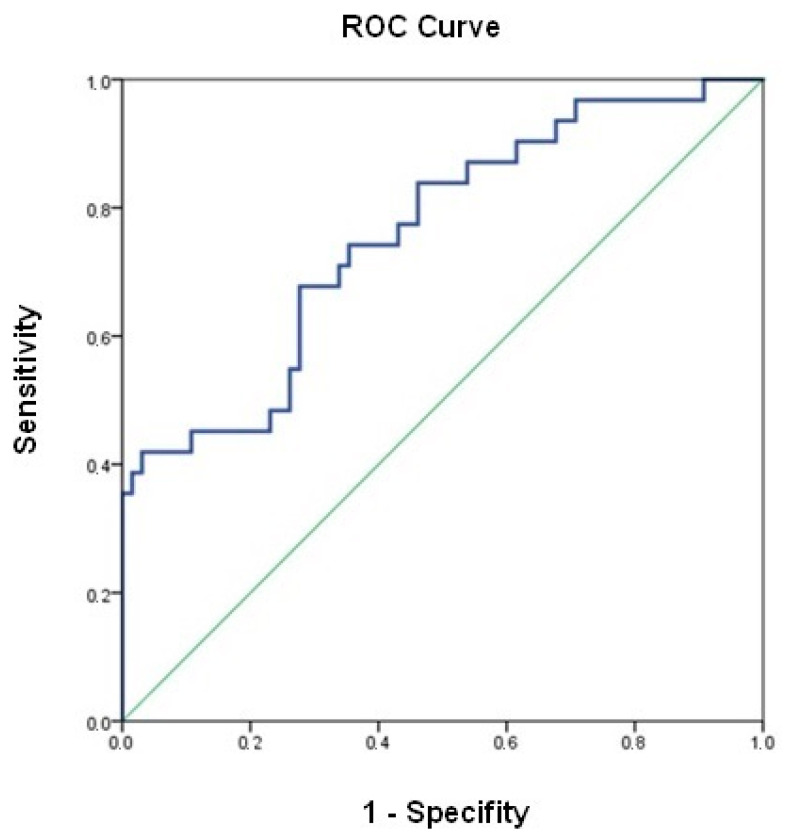
ROC curve for the multivariable Cox regression model.

Univariate analysis identified previous oral furosemide doses (HR 2.85 [1.01–8.07]; *p* = 0.049), eGFR at admission (HR 0.39 [0.18–0.86]; *p* = 0.020), admission urinary sodium concentrations > 80 mmol/L (HR 0.46 [0.24–0.90]; *p* = 0.023), and admission NT-proBNP concentrations (HR 1.43 [1.07–1.90]; *p* = 0.016) as potential predictors for the primary outcomes. After adjusting for seven variables, the multivariate Cox regression analysis identified urinary sodium concentration > 80 mmol/L (HR 0.50 [0.25–1.02]; *p* = 0.056) and initial CA125 concentrations (HR 1.44 [0.98–2.10]; *p* = 0.073) as independent risk predictors for the primary endpoint. The area under the curve for that model was 0.759 (0.654–0.862) (*p* < 0.001). (Table 3 and Figure 3).

## 4. Discussion

The main findings of this study were that an optimal diuretic response after initial e.v. loop diuretics (NaU > 80 mmol/L) was associated with effective decongestion with less pulmonary and intravascular congestion and a significant fall of IAP. This behavior of IAP has not been described previously. Notably, an insufficient diuretic response (NaU ≤ 80 mmol/L) was frequent (40% of the cohort), and we independently predicted ACM and/or HF rehospitalizations at 90 days.

### 4.1. Natriuresis and Decongestion in Acute Heart Failure

Congestion is the primary therapeutic target in ADHF patients [1]. Currently, HF guidelines recommend the use of e.v. loop diuretics for symptomatic relief in ADHF, adjusting the initial doses based on previous furosemide oral intake [1]. Despite the awareness of the decongestive efficacy of loop diuretics, the reality is that up to one-third of the patients discharged after an episode of ADHF still have subtle signs and/or symptoms of congestion, so-called “persistent congestion”, leading to worse outcomes [4]. Consequently, during the last decade, efforts have been focused on the search for efficient treatment strategies to improve the schedule of diuretics dosage aimed to remove residual congestion at discharge. Several strategies adding biomarker-guided therapy have been used to address this issue. In the CHANCE-HF study [8], CA125 concentrations on admission were used to adjust the initial e.v. loop diuretic doses. In the LUS-HF trial [28], tailored lung-ultrasound-guided diuretic treatment reduced the number of decompensations and improved functional status in outpatient HF patients. More recently, the analysis of urinary metrics, especially natriuresis, has shown promising results in guiding current strategies for the adjustment of diuretics dosage. They are based on the natriuretic response to the initial doses of loop diuretics administered early after admission.

In our study, up to 40% of patients had a poor diuretic response (NaU ≤ 80 mmol/L) associated with impaired prognosis during the first 90 days after discharge. These results are similar to that of the study by Verbrugge et al. [20], which was a posthoc analysis of the cohort of ADVOR clinical trial [29] (Acetazolamide in Decompensated Heart Failure with volume overload). The authors found that patients with an insufficient diuretic response had a worse prognosis due to an increase in death or readmission for HF.

Our results support the additional value of multimodal assessment of congestion performed early after admission. To date, most of the published studies rely on the assessment of the natriuresis from a spontaneous urine sample and congestion assessed either through a physical examination (congestion scores) or serum biomarkers (NT-proBNP or CA125) [11,12,30].

In our cohort, the assessment of congestion was addressed with a multimodal approach that included ultrasounds, serum biomarkers, the natriuretic response, and, for the first time, the measurement of IAP. All these parameters were evaluated at baseline and 48 h after the initial doses of loop diuretics had been administered. We did not find differences in congestion at baseline, but those patients with a good natriuretic response (>80 mEq/L) showed a clear trend of decongestion in terms of in ultrasound and through biomarkers, and they had a significant fall in IAP and fewer outcomes than those with natriuresis below that level. In addition, urine Na concentration continued to be higher in patients with an initial good response.

The change in IAP early after diuretic administration deserves a comment. Abdominal congestion [31,32] has been described as an important pathophysiological mechanism for the development of congestive nephropathy. So far, our study is the first to show that an optimal diuretic response (NaU > 80 mmol/L) is associated with a significant reduction in IAP during the first 48 h of admission. Unfortunately, this measurement was available in only 57 patients, which limits the generalization and interpretation of our results. Even though it seems plausible that natriuresis and reduction in IAP, induced by the initial doses of loop diuretics, allow clinicians to identify the subgroup of patients more prone to residual congestion, and whether the intensification of diuretics in this group is beneficial should be tested in adequately designed studies.

### 4.2. Clinical Implications

Our results agree with other retrospective analyses on urinary metrics and diuretic response [33,34]. Together, they suggest that these parameters will play a much more relevant role in future HF guidelines. Indeed, two ongoing clinical trials are testing such a strategy, the ENACT-HF [35] clinical trial and the PUSH-HF [18] clinical trial, whose final results are expected in the upcoming months.

What is clear is that achieving an optimal diuretic response as quickly as possible after admission is crucial to improving the prognosis of our patients with decompensated HF. In this context, the combined diuretic strategy will probably become more prominent, as the ADVOR [29] (e.v. furosemide plus acetazolamide) and CLOROTIC [36] (furosemide plus hydrochlorothiazide) trials have shown.

## 5. Conclusions

Poor diuretic response is common in patients admitted for ADHF and is associated with a higher degree of lung and intravascular residual congestion and a higher risk of mortality after discharge. Patients with an optimal diuretic response achieve decongestion more easily, as is shown by the reduction in the IVC diameter and IAP 48 h after admission. Natriuresis 2 h after initiation of e.v. loop diuretics and the change in IAP 48 h after admission seem to be feasible and valuable tools to identify diuretic response and residual congestion in ADHF. Both tests should probably be implemented more frequently in clinical settings.

## 6. Limitations

The study has several limitations. First, it is a retrospective study with a small sample size that limits statistical power. Second, natriuresis was measured the morning following admission to the Internal Medicine ward; thus, most patients had received some dose of e.v. furosemide in the Emergency department, which could have reduced the power of the study. Third, the analysis of IAP is limited to only 57 patients due to the difficulty of obtaining it in the currently overloaded clinical departments. Also, patients with contraindications for bladder catheterization were not assessed for this parameter. Despite this, the study should try to overcome such barriers in the future.

## Figures and Tables

**Table 1:** Baseline characteristics according to sodium urinary concentrations at admission.

Variable	Total	NaU ≤ 80	NaU > 80	*p*-Value
N (%)	113 (100)	45 (39.8)	68 (60.2)	
Age (years)	81.7 ± 8.3	82.0 ± 7.3	81.5 ± 9.0	0.727
Women (%)	61 (54.0)	22 (19.5)	39 (57.4)	0.377
NYHA				0.159
NYHA I (n [%])	20 (17.7)	6 (13.3)	14 (20.6)	
NYHA II (n [%])	65 (57.5)	24 (53.3)	41 (60.3)	
NYHA III (n [%])	26 (23.0)	13 (28.9)	13 (19.1)	
NYHA IV (n [%])	2 (1.8)	2 (4.4)	0 (0.0)	
LVEF				0.434
HFpEF (n [%])	58 (61.1)	19 (52.8)	39 (66.1)	
HFmrEF (n [%])	13 (13.7)	6 (16.7)	7 (11.9)	
HFrEF (n [%])	24 (25.3)	11 (30.6)	13 (22.0)	
Hypertension (n [%])	92 (81.4)	33 (73.3)	59 (86.8)	0.072
Diabetes (n [%])	41 (36.3)	13 (28.9)	28 (41.2)	0.184
AF (n [%])	75 (66.4)	30 (66.7)	45 (66.2)	0.957
COPD (n [%])	22 (19.5)	13 (28.9)	9 (13.2)	0.040
ICD (n [%])	30 (26.5)	16 (35.6)	14 (20.4)	0.078
Dyslipidemia (n [%])	62 (54.9)	25 (55.6)	37 (54.4)	0.905
eGFR (mL/min)	51.6 (32.8)	47.2 (25.9)	58.1 (56.1)	0.096
eGFR groups (n [%])				0.054
eGFRv ≥ 60 (n [%])	36 (36.0)	10 (25.6)	26 (42.6)	
eGFR 30–59 (n [%])	49 (49.0)	25 (64.1)	24 (39.3)	
eGFR < 29 (n [%])	15 (15.0)	4 (4.0)	11 (18.0)	
HF treatment				
ACEi/ARB (n [%])	67 (59.3)	25 (55.6)	42 (61.8)	0.511
Sacubitril/valsartan (n [%])	4 (3.5)	2 (4.4)	2 (2.9)	0.672
B-Blockers (n [%])	69 (61.1)	28 (62.2)	41 (60.3)	0.837
MRB (n [%])	24 (21.2)	9 (20.0)	15 (22.1)	0.793
SGLT2i (n [%])	10 (8.8)	5 (11.1)	5 (7.4)	0.491
Diuretics				
Furosemide (n [%])	86 (76.1)	40 (88.9)	46 (67.6)	0.010
Outpatient furosemide dose (mg)	40 (55)	40 (53)	35 (40)	0.049
HCTZ (n [%])	22 (19.5)	10 (22.2)	12 (17.6)	0.549
Laboratory				
NT-proBNP (pg/mL)	4898 (10,630)	6227 (13,835)	4113 (5384)	0.056
CA125 (pg/mL)	38.1 (49.3)	37.6 (44.9)	38.7 (52.2)	0.766
Hemoglobin (g/L)	11.8 ± 1.8	11.8 ± 1.8	11.7 ± 1.8	0.778
Sodium (mmol/L)	140 ± 4	138 ± 5	141 ± 3	<0.001
Potassium (mmol/L)	4.1 ± 0.5	4.1 ± 0.5	4.1 ± 0.5	0.828
Chloride (mmol/L)	99 ± 6	97 ± 6	100 ± 4	0.002

NYHA: New York Heart Association; LVEF: left ventricular ejection fraction; HFpEF: heart failure with preserved ejection fraction; HFmrEF: heart failure with mildly reduced ejection fraction; HFrEF: heart failure with reduced ejection fraction; AF: atrial fibrillation; COPD: chronic obstructive pulmonary disease; ICD: ischaemic coronary disease; eGFR: estimated glomerular filtration rate; HF: heart failure; ACEi: angiotensin-converting enzyme inhibitors; ARB: angiotensin receptor blockers; MRB: mineralocorticoid receptor blockers; iSGLT2: sodium-glucose co-transporter-2 inhibitors; HCTZ: hydrochlorothiazide; NT-proBNP: N-terminal pro b-type natriuretic peptide; CA125: cancer antigen 125.

**Table 2:** Markers of congestion and intraabdominal pressure level, according to initial urine sodium concentrations.

Variable	Baseline			Control at 48 h		
	NaU ≤ 80	NaU > 80	*p*-Value	NaU ≤ 80	NaU > 80	*p*-Value
B-lines by LUS (n)	21 (14)	21 (12)	0.927	15 (17)	12 (14)	0.084
IVC diameter (mm)	21 (7)	22 (7)	0.973	22 (6)	18 (5)	0.009
IVC colapsability < 50%	38 (86.4)	49 (75.4)	0.161	32 (72.7)	38 (59.4)	0.153
IAP (mmHg)	15 (6)	14 (5)	0.473	13 (6)	11 (5)	0.041
Natriuresis (mmol/L)				75 (43)	84 (55)	0.042
Total IV furosemide dose (mg)				180 (138)	140 (80)	0.100

LUS: Lung ultrasound; IVC: inferior vena cava; IAP: intraabdominal pressure; IV: intravenous; NaU: urine sodium.

**Table 3:** Univariable and multivariate Cox regression analysis for the primary endpoint (all-cause mortality and/or HF rehospitalizations at 90 days).

	Univariable		Multivariate	
Variable	HR (CI 95%)	*p*-Value	HR (CI 95%)	*p*-Value
Age (years)	1.02 (0.97–1.06)	0.462		
Gender (male)	1.49 (0.76–2.89)	0.243		
LVEF (%)	0.57 (0.20–1.59)	0.285		
SBP (mmHg)	0.99 (0.98–1.01)	0.265		
DBP (mmHg)	0.99 (0.97–1.01)	0.382		
HR (B.p.m.)	1.01 (0.99–1.03)	0.265		
HTA	0.71 (0.32–1.57)	0.402		
Diabetes	1.04 (0.52–2.06)	0.916		
COPD	1.75 (0.82–3.74)	0.147		
AF	1.05 (0.52–2.15)	0.887		
CID	1.26 (0.62–2.58)	0.521		
Dislipidemia	1.90 (0.93–3.89)	0.077		
Previous use of furosemide	2.85 (1.01–8.07)	0.049		
Previous use of thiazides	1.21 (0.55–2.66)	0.642		
Previous use of MRB	1.55 (0.75–3.24)	0.239		
Previous use of b-blocker	1.65 (0.79–3.44)	0.182		
Previous use of ACEi/ARB	1.15 (0.58–2.28)	0.690		
Previous use of SGLT2i	1.08 (0.38–3.05)	0.890		
Egfr *	0.39 (0.18–0.86)	0.020		
Natriuresis > 80 mEq/L	0.46 (0.24–0.90)	0.023	0.50 (0.25–1.02)	0.056
B-lines at baseline *	1.01 (0.57–1.82)	0.963		
B-lines at 48 h *	0.99 (0.58–1.69)	0.981		
IVC diameter at baseline *	1.02 (0.31–3.38)	0.979		
IVC diameter at 48 h *	2.91 (0.89–9.51)	0.078		
IAP at baseline *	0.73 (0.18–2.97)	0.663		
IAP at 48 h *	2.91 (0.72–11.8)	0.135		
NT-proBNP at baseline *	1.43 (1.07–1.90)	0.016		
CA125 at baseline *	1.40 (0.97–2.04)	0.073	1.44 (0.98–2.10)	0.059
Haemoglobin at baseline	0.90 (0.75–1.07)	0.242		

LVEF: Left ventricular ejection fraction; SBP: systolic blood pressure; DBP: diastolic blood pressure; HR: heart rate; HTA: hypertension; COPD: chronic obstructive pulmonary disease; AF: atrial fibrillation; CID: coronary isqueamic disease; MRB: mineralocorticoid receptor blockers; ACEi: angiotensin-converting enzyme inhibitors; ARB: angiotensin receptor blockers; iSGLT2: sodium-glucose co-transporter-2 inhibitors; eGFR: estimated glomerular filtration rate; IVC: inferior vena cava; IAP: intraabdominal pressure; NT-proBNP: N-terminal pro b-type natriuretic peptide; CA125: cancer antigen 125. NT-proBNP: Model was adjusted using NT-proBNP at baseline; CA125 at baseline; IVC diameter after 48 h; urinary sodium concentrations at baseline; eFGR; previous intake of loop diuretics; history of dyslipidemia. AUC of the model 0.759 (0.654–0.862); *p*-value < 0.001. * variables have been transformed using fractional polynomials.

## Data Availability

The data that support the findings of this study are not publicly available due to their containing information that could compromise the privacy of research participants, but they are available from JRG (corresponding author).
